# Monitoring the impacts of rainfall characteristics on sediment loss from road construction sites

**DOI:** 10.1007/s11356-024-33361-3

**Published:** 2024-04-23

**Authors:** Alec G. Grimm, Richard A. Tirpak, Ryan J. Winston

**Affiliations:** 1 Dept. of Food, Agricultural, and Biological Engineering, The Ohio State University, 590 Woody Hayes Dr, Columbus, OH 43210 USA; 2 Dept. of Civil, Environmental, and Geodetic Engineering, The Ohio State University, 2070 Neil Ave, Columbus, OH 43210 USA

**Keywords:** Construction site runoff, Erosion and sediment control measures, Stormwater, Particle size distribution, Erosion, Water quality

## Abstract

Exposed soils associated with active construction sites provide opportunities for erosion and sediment transport during storm events, introducing risks associated with excess sediment to downstream infrastructure and aquatic biota. A better understanding of the drivers of sediment transport in construction site runoff is needed to improve the design and performance of erosion and sediment control measures (ESCMs). Eleven monitoring locations on 3 active road construction sites in central Ohio were established to characterize runoff quality from points of concentrated flow during storm events. Grab samples were analyzed for total suspended solids (TSS), turbidity, and particle size distribution (PSD). Median TSS concentrations and turbidity levels across all samples were 626 mg/L (range 25–28,600 mg/L) and 759 NTU (range 22–33,000 NTU), respectively. The median PSD corresponded to a silty clay loam, mirroring the soil texture of much of Ohio’s subsoils. TSS concentrations and turbidity were significantly positively correlated with the rainfall intensity 10 min prior to sample collection, suggesting that higher flow rates created greater shear stress on bare soil which resulted in more erosion. Conversely, rainfall duration was negatively correlated with particle size, indicating that prolonged moisture from rainfall promoted the dispersion of soil aggregates which mobilized smaller particles. Multivariable linear regression models revealed that higher rainfall intensities corresponded to higher turbidity values, while higher TSS concentrations were associated with higher rainfall intensities, depths, and durations. Results from this study highlight the importance of reducing raindrop impact and subsequent shear stress applied by concentrated flows through the use of ESCMs to limit sediment export from construction sites.

**Supplementary Information:**

The online version contains supplementary material available at 10.1007/s11356-024-33361-3.

## Introduction

Construction activities involve mass grading, excavation, and removal of existing vegetation, exposing bare soil to erosive elements (i.e., wind and rain). Exposed soil can be eroded during storm events and transported offsite into receiving waterbodies, adversely impacting aquatic ecosystems (Barron 1995; Wood and Armitage 1997; Langston et al. 2010). Impacts include reduced clarity in the water column and subsequent effects on predator–prey interactions (Fiksen et al. 2002; Lunt and Smee 2014), acute or chronic toxicity to organisms from particulate-bound pollutants (e.g., polycyclic aromatic hydrocarbons, polychlorinated biphenyls, heavy metals, and excess nutrients; Barron 1995; Langston et al. 2010), smothering of benthic habitat (Wood and Armitage 1997; Lalor et al. 2004), and reduced photosynthetic output from aquatic vegetation (Lloyd et al. 1987). Excessive sediment loading also impacts existing infrastructure by reducing the capacity of channels, storm sewer networks, and reservoirs (Crowder 1987; Rahmani et al. 2018) and accelerates the clogging of infiltration-based stormwater control measures such as permeable pavements and bioretention cells (Blecken et al. 2017; Tirpak et al. 2021; Winston et al. 2016).

Sediment loads from construction sites are often tens to hundreds of times greater than those from agricultural lands (Daniel et al. 1979; Santikari and Murdoch 2019; Wolman and Shick 1967) and thousands of times greater compared to urban areas and forests (Line et al. 2002; Simpson et al. 2022). Thus, mitigating sediment export from construction sites is critical to maintaining ecosystem health and has been the focus of research for over five decades. Regulations developed under the Clean Water Act and National Pollutant Discharge Elimination System permits require construction activities in the USA which disturb more than 0.4 ha of land to implement a stormwater pollution prevention plan (SWPPP). SWPPPs aim to limit the amount of sediment and pollutants discharged into receiving waters and storm sewer networks during construction (USEPA 2000). This is often accomplished using erosion and sediment control measures (ESCMs) (e.g., sediment basins, erosion control blankets, inlet protection, check dams, vegetation establishment) to retain sediment on-site.

Drainage area, soil type, rainfall intensity, and sediment loading rate are the primary factors considered in the design of sediment basins, inlet protection, and silt fence (Zech et al. 2014; Perez et al. 2015; Bugg et al. 2017). Soil loss equations, including the universal soil loss equation (USLE), modified universal soil loss equation (MUSLE), and revised soil loss equation (RUSLE), are also an integral part of the design of ESCMs. These equations utilize many factors such as soil type, rainfall, slope, and vegetative cover to predict expected soil losses from erosion. However, these models do not rely on site-specific conditions (i.e., soil properties) and may underestimate soil losses (Clark et al. 2009). It is also noted that the rainfall erosivity factor (i.e., R-factor) used in soil loss equations varies highly by region due to varying rainfall and soil characteristics (Ebrahimzadeh et al. 2018). The R-factor is a function of the total kinetic energy of precipitation and the maximum rainfall intensity over a 30-min period (Brown and Foster 1987). However, this value may vary across construction sites due to rainfall patterns and site-specific conditions (Kinnell 1973). Therefore, an improved understanding of the drivers of sediment production on active construction sites, which can ultimately aid in the design and implementation of ESCMs, is needed (Pitt et al. 2007).

Sediment export from construction sites has been the subject of numerous studies (Wolman and Schick 1967; Daniel et al. 1979; Line et al. 2002, 2011; Line and White 2007); however, few have analyzed the effect of rainfall characteristics on total suspended solids (TSS), turbidity, and particle size distribution (PSD) in construction site runoff, particularly when no ESCMs are in place (i.e., prior to treatment). Table 1 outlines the range of TSS and turbidity values reported by previous studies of construction site runoff in North America.

**Table 1:** Summary of TSS and turbidity values from active construction sites in previous research

Study	Location	TSS range (mg/L)	Turbidity range (NTU)
McLaughlin et al. (2009)	North Carolina, USA	870–7760	3100–11,480
Binns et al. (2019)	Ontario, Canada	538–34,000	NA
Schussler et al. (2022)	Alabama, USA	NA	669–6781
Fang et al. (2015)	Alabama, USA	95–26,325	191–28,352
Smith (2018)	Tennessee, USA	10,000–165,000	NA
Schussler et al. (2020)	Iowa, USA	2–4007	43–6781

Previous research has demonstrated that TSS and turbidity levels are highly variable in construction site runoff. Several factors impact sediment transport and erosion potential in soils (e.g., soil type, soil water content, land use, rainfall characteristics, flow velocity, vegetative cover, slope; Renard and Ferreira 1993; Sear 1996; Römkens et al. 2002). Kayhanian et al. (2001) demonstrated that site-specific soil properties and the presence of vegetation could explain this variability; conversely, Daniel et al. (1979) attributed varying flow characteristics to the wide range of TSS concentrations detected in construction site runoff. Similarly, Shen et al. (2018) found that turbidity levels tended to increase following the onset of rainfall before gradually declining following the cessation of rainfall; rainfall depth and peak turbidity were also positively correlated. Given this lack of consensus, an improved understanding of the factors (e.g., rainfall characteristics, site conditions) which influence the quantity and characteristics (e.g., PSD) of sediment exported from construction sites is needed to improve the design and implementation of ESCMs.

Particle size distribution in post-construction highway runoff has also been studied extensively (Sansalone et al. 1998; Furumai et al. 2002; Li et al. 2005; Charters et al. 2015; Selbig et al. 2016; Hilliges et al. 2017; Winston and Hunt 2017; Winston et al. 2023). However, such relationships between rainfall, soil, and site-specific factors and PSDs in construction site runoff have not yet been explored for active construction sites. Particle size distribution of construction site runoff is crucial to the design of ESCMs, particularly those that function by sedimentation (e.g., sediment basins and silt fence; Greb and Bannerman 1997; Nighman and Harbor 1997; Keener et al. 2007). Particle size distributions also vary due to biological, chemical, and physical soil composition, which differ widely across the world (Amundson et al. 2003). Thus, the effects of rainfall characteristics (i.e., depth, intensity, duration) may have vastly different impacts on PSD (and thus TSS and turbidity levels) in construction site runoff in different regions.

It is widely theorized that the rate of soil particle detachment is related to rainfall intensity (Mahmoodabadi and Sajjadi 2015), with higher rainfall intensities supplying enough energy to dislodge larger particles. However, the impacts of rainfall characteristics on particle size may vary widely given the spatial variation of soil properties. For example, Martínez-Mena et al. (2002) found that rainfall intensity had no impact on the PSD of a silt loam soil due to surface crusting properties but observed a decrease in coarser fractions with longer runoff time in a different silt loam soil. Land slope can also affect PSD in runoff (Wischmeier and Smith 1958); crucially, while all particle size fractions are impacted by rainfall intensity, land slope has been shown to have less of an effect on smaller particles (i.e., clay and coarser silt) compared to rainfall intensity (Kiani-Harchegani et al. 2019). Clearly, varying degrees of soil properties influence particle transport in runoff. However, studies investigating the relationship between rainfall and PSD in construction site runoff have yet to be performed. Thus, research is needed to understand the drivers of erosion and sediment production on construction sites as it relates to rainfall.

This study presents the results of a 13-month field monitoring campaign at three active highway construction sites across central Ohio, USA. The objective of this study was to (1) determine how rainfall characteristics influence TSS, turbidity, and PSD on active highway construction sites and (2) assess the variability in sediment characteristics from multiple runoff-producing storm events. Results from this research be used to inform the design and implementation of ESCMs to mitigate sediment export from active construction projects.

## Materials and methods

### Study description

**Fig. 1:**
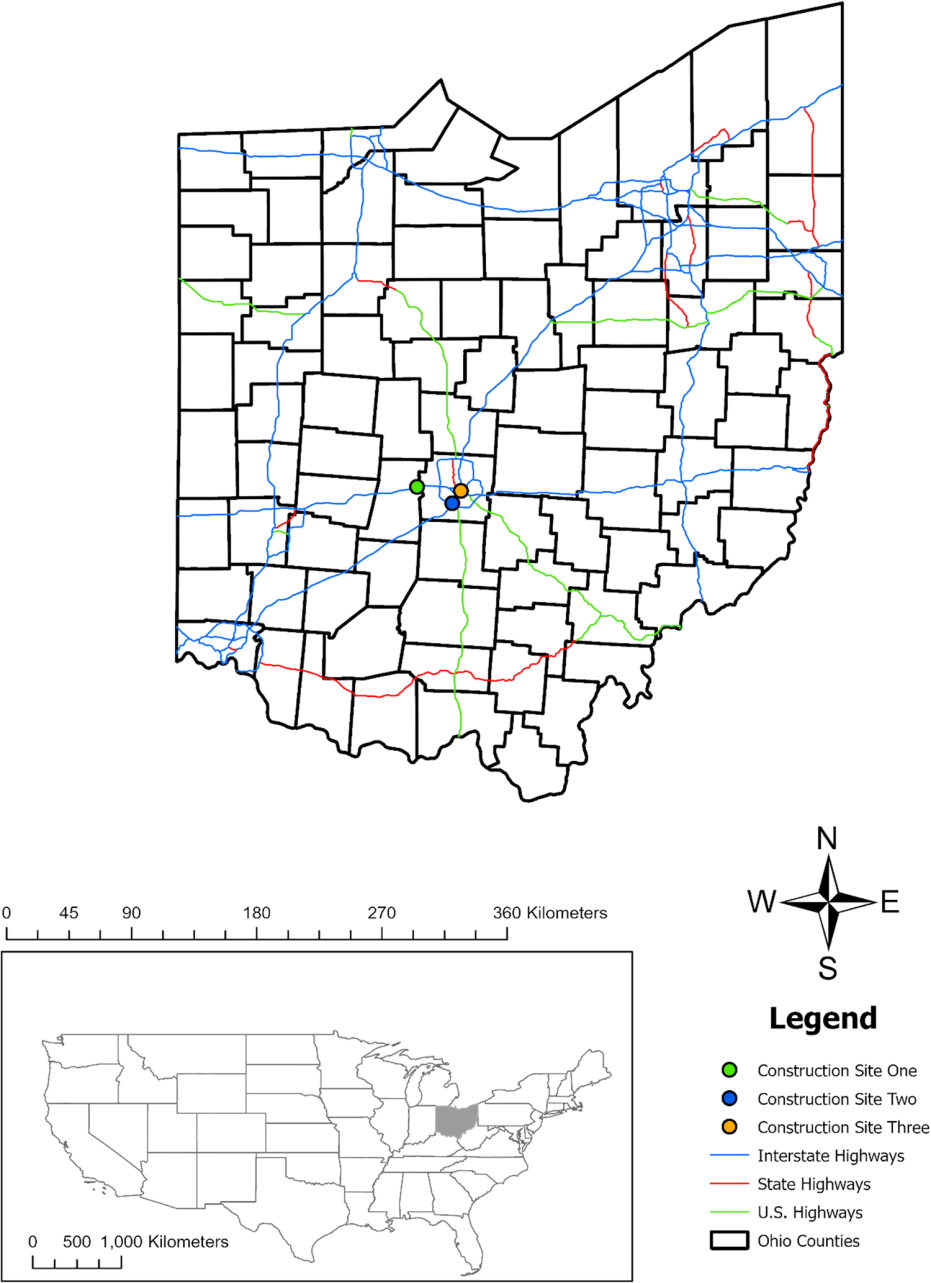
Location of three active construction sites where runoff samples were collected

Field monitoring was conducted between 2019 and 2020 at 11 monitoring locations at three active interstate highway construction sites during different construction phases in central Ohio, USA (Fig. 1). All sites contained exposed soil typical of active construction and were selected to investigate the typical sediment characteristics in untreated construction site runoff. Site 1 involved the reconstruction and widening of the inside shoulders along Interstate 70 in Madison and Franklin counties, Ohio. Site 2 was a major rehabilitation of Interstate 71 near Grove City, Ohio. At site 3, 1.2 km of Interstate 70 was reconfigured and reconstructed near downtown Columbus, Ohio.

Monitoring locations located within the same construction site were differentiated by drainage outlets and located in areas of concentrated flow. As construction projects advanced through different phases, considerable changes in drainage behavior associated with earth-moving activities occurred at each monitoring location. In these instances, a new location was chosen if drainage area land cover changed substantially from original conditions (i.e., application of straw or grass seed or installation of asphalt). The number of samples collected and the number of storm events sampled by monitoring location as well as the description of each monitoring location are presented in Table 2.

**Table 2:** Description of monitoring locations

Construction site	Monitoring location	Latitude	Longitude	Storm events sampled	Number of samples	Period sampled	Description
One	A	39.976476	− 83.261082	1	5	October 2019	Near construction entrance
One	B	39.977821	− 83.259513	1	5	October 2019	Ditch next to highway
Two	C	39.880092	− 83.047741	1	5	October 2019	Ditch next to highway onramp
Two	D	39.88995	− 83.038705	5	21	October 2019–May 2020	Upstream of rock ditch check
Three	E	39.954714	− 82.980449	2	8	January 2020	Bare road section
Three	F	39.954646	− 82.978062	2	8	January 2020	Bare road section
Three	G	39.954918	− 82.981005	1	5	November 2020	Bare road section under bridge bypass
Two	H	39.887796	− 83.042482	2	13	November 2020	Ditch upstream of riprap and check dam
One	I	39.978878	− 83.260687	1	5	May 2020	Bare earth section on off ramp
Two	J	39.881304	− 83.047345	5	23	September 2020–November 2020	Ditch upstream of silt fence ditch check
Three	K	39.954714	− 82.980449	2	6	May 2020–August 2020	Bare road section

### Sample collection

Grab samples were collected from areas of concentrated flow to characterize sediment in runoff at each construction site. Areas of concentrated flow were typically located within existing ditches and upstream of sediment and erosion controls. Crucially, samples were collected from areas lacking vegetation (i.e., bare soil) and treatment from sediment and erosion controls to best characterize untreated runoff from construction sites. Samples were collected at approximately 1-h intervals or in approximately equal intervals over the duration of the storm event due to travel time between monitoring locations during rain events. To fill the sample bottle during periods of lower flows, small aliquots were collected in the sample bottle lid (approx. 50 mL) and transferred to the sample bottle until a 1 L composite sample volume was obtained. Higher resolution sampling (i.e., every 60 min) was conducted for a storm event on November 25, 2020, at monitoring locations G, H, and J to understand how sediment characteristics changed over the course of a storm hyetograph.

Rainfall was monitored at each construction site using 0.254-mm resolution Davis Rain Collector tipping bucket rain gages (Davis Instruments, Hayward, California) installed in areas free from overhead obstructions. Data were stored at 1-min intervals using Hobo Pendant data loggers (Onset Computer Corporation, Bourne, Massachusetts) and downloaded monthly. Storm events were separated by a minimum 6-h antecedent dry period (ADP) and a minimum rainfall depth of 2.5 mm. If data could not be retrieved after a storm event (i.e., due to a clogged rain gage inlet, dead battery, or frozen bucket), rainfall data was downloaded at 5-min intervals from a nearby gage within 5 km of the sample site; this occurred four times over the course of the study, affecting 26 samples.

### Sample analysis

Grab samples were stored at approximately 2 °C within 6 h of sample collection. Approximately 100 mL aliquots from each grab sample were used for TSS analysis after thoroughly mixing each sample to provide a representative subsample for subsequent laboratory analysis. TSS was then determined by vacuum filtration using ASTM method D5907-18 (ASTM D5907-18 2018; see supplementary materials Fig. 1A). Turbidity was measured using a HACH 2100q portable turbidimeter (0–1000 NTU) (see supplementary materials Fig. 1A). Turbidity values were only obtained for 9 of the 13 sampled storm events (67 out of 104 total grab samples). Before turbidity readings were taken, the sample cell was thoroughly shaken to ensure a uniform distribution of particles. Samples which exceeded the maximum turbidity of the instrument were diluted using DI water. Once within the range of the turbidimeter, readings were multiplied by the dilution factor to determine the actual turbidity of the sample using the following equation:

1 $${\text{Turbidity}}={T}_{D }\times \frac{{V}_{t}}{{V}_{s}}$$

where *T*_*D*_ is the turbidity reading of the diluted sample (NTU), *V*_*t*_ is the total volume of the original sample with DI water added (mL), and *V*_*s*_ is the volume of the original sample (mL). Five turbidity measurements were made for samples requiring dilution; the average was used for the reported turbidity value herein. Three readings were performed for samples that did not require dilution, with the average reported as the final turbidity value.

Particle size distributions were determined using a Beckman Coulter LS 13–320 laser diffraction particle size analyzer capable of characterizing particle sizes in 117 particle diameter channels ranging from 0.04–2000 μm (see supplementary materials Fig. 1A). Samples were refrigerated at approximately 2 °C for at least 1 week prior to analysis to allow for settling to occur within the sample. Approximately 5 mL of the settled sediment was then pipetted into the analyzer to determine the volumetric percentage of particles across the 117 particle diameter channels.

### Data analysis

Summary statistics including rainfall depth (mm); duration (h); peak 5-min rainfall intensity (mm/h); average intensity (mm/h); the 10-, 30-, 60-, and 120-min rainfall intensity (mm/h) prior to obtaining the grab sample; and ADP (days) were determined for all sampled rainfall events. Results from PSD analyses were used to determine the 10th, 50th, 60th, and 90th percentile particle diameter (i.e., d_10_, d_50_, d_60_, d_90_) as well as the mean particle diameter and uniformity coefficient (*C*_*u*_) for each sample. The *C*_*u*_ was calculated using Eq. 2:

2 $${C}_{u}=\frac{{{\text{d}}}_{60}}{{{\text{d}}}_{10}}$$

Spearman’s rank correlation was used to identify significant correlations between PSD, TSS, turbidity, and rainfall characteristics. To predict TSS and turbidity values generated from construction site runoff, backward selection multivariable linear regression (MLR) models were created using rainfall and PSD characteristics as explanatory variables. The Durbin-Watson test was used to test for autocorrelation among the MLR models. Assumptions of normality were confirmed graphically using Q-Q plots. All statistical analyses were performed in Excel (Microsoft) and R version 3.6.2 (R Core Team 2019); *p*-values less than 0.05 were considered statistically significant.

## Results and discussion

### Observed rainfall characteristics

**Fig. 2:**
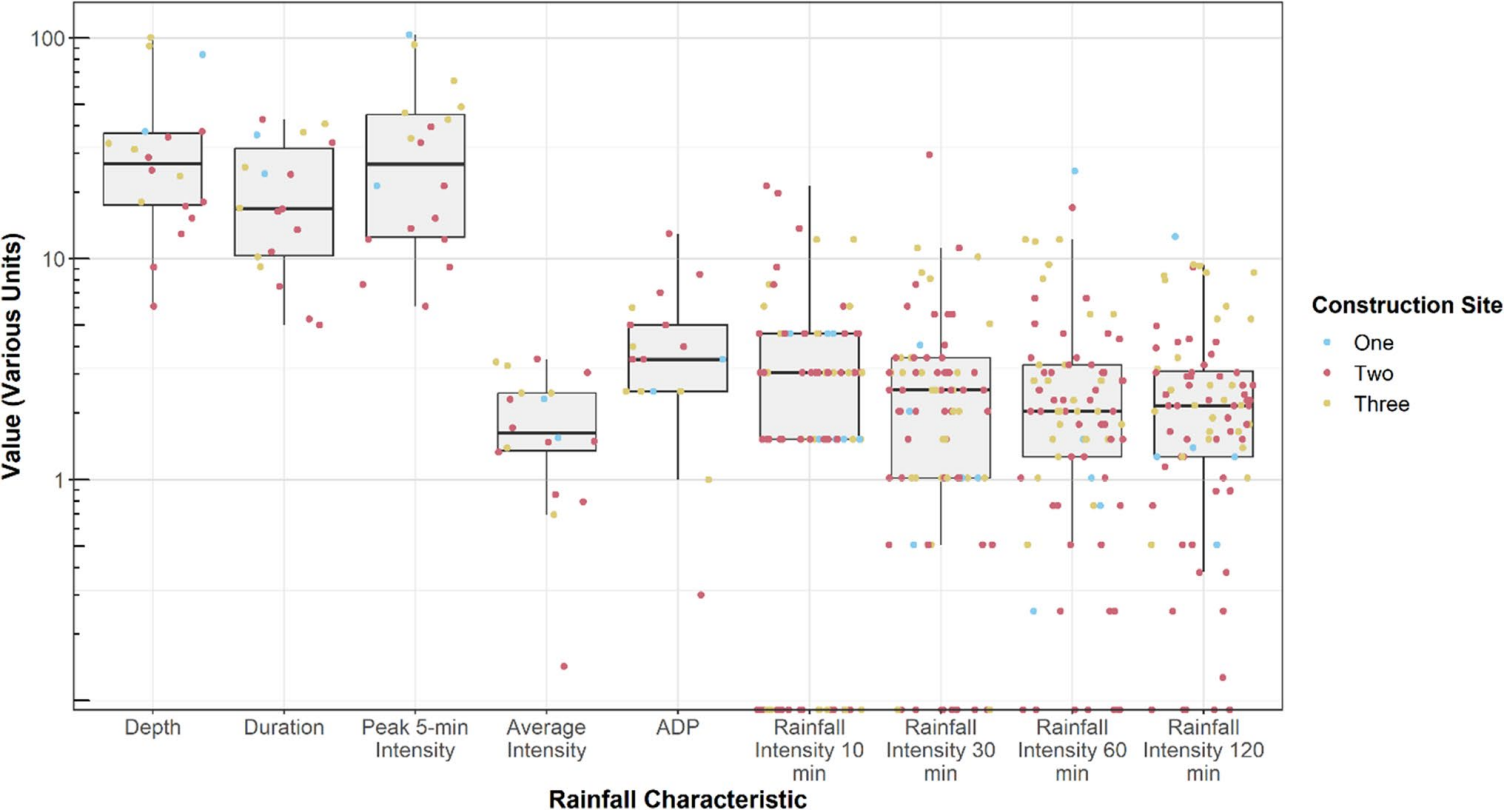
Boxplots of 10-, 30-, 60-, and 120-min rainfall intensity (mm/h), depth (mm), duration (h), peak intensity (mm/h), average intensity (mm/h), and antecedent dry period (ADP, days). Note: rainfall characteristics plotted on a log scale

A total of 18 storm events were monitored between October 2019 and November 2020. Substantial variability in rainfall characteristics was observed during the monitoring period (Fig. 2). Rainfall depths ranged from 6.1 to 100.3 mm with a median depth of 27 ± median absolute deviation (MAD) of 10 mm. Peak 5-min and average rainfall intensities varied from 6.1 to 93 mm/h (median and MAD of 27 ± 15 mm/h) and 0.1 to 3.5 mm/h (median and MAD of 1.6 ± 1 mm/h), respectively. Rainfall duration and ADP exhibited similar trends, ranging from 5 to 43 h (median and MAD of 17 ± 8 h) and 0.3 to 13 days (median and MAD of 3.5 ± 1 day), respectively. Median peak 5-min and average rainfall intensities observed during this study were less than historic rainfall records (i.e., 1949–2016) for central Ohio (USGS 2016), while rainfall duration and ADP were greater than historical averages.

### TSS and turbidity

**Fig. 3:**
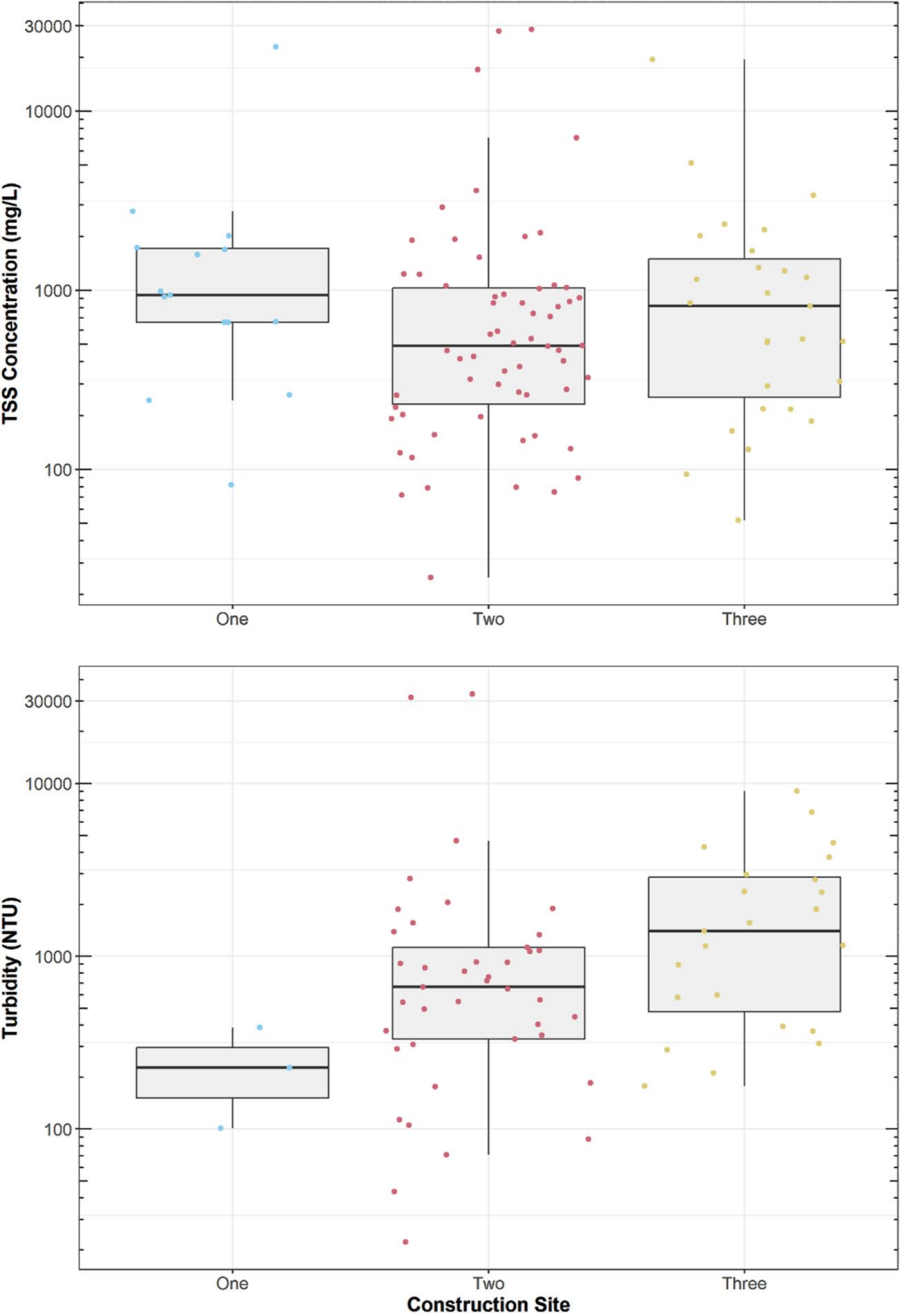
Distribution of TSS concentrations and turbidity values across each construction site. Note: parameters are plotted on log scales

A total of 104 samples were analyzed for TSS across 18 storms during the study. Substantial variability was observed in both TSS concentrations and turbidity levels (Fig. 3). TSS concentrations ranged from 25 to 28,600 mg/L (median of 626 mg/L ± MAD 426 mg/L). Similarly, turbidity values ranged from 22 NTU to 33,000 NTU (median of 759 NTU ± MAD 541 NTU). High variability among TSS and turbidity was also observed in previous studies of construction site runoff (Table 1), suggesting that substantial variation in sediment export should be expected during construction. While runoff hydrology was not measured in this study, the wide range of rainfall characteristics observed (Fig. 2) suggests that a broad range of flow characteristics occurred, potentially explaining the variation in sediment export. These observations are consistent with Daniel et al. (1979) and Walling and Gregory (1970), who found that most of the variation observed in sediment loading was related to varying runoff volumes.

**Fig. 4:**
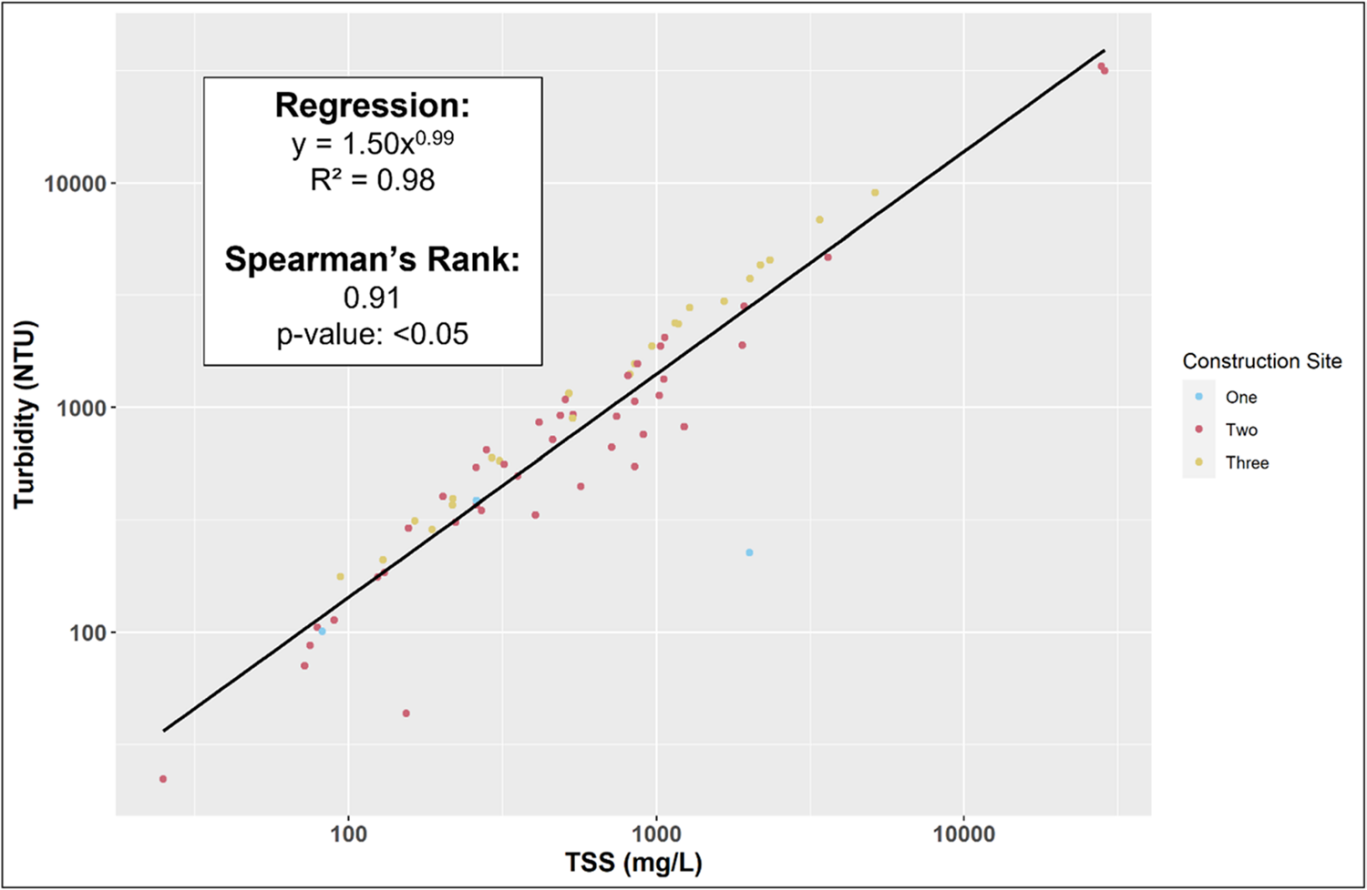
Relationship between turbidity and TSS

TSS and turbidity were significantly correlated (Fig. 4). Turbidity is dependent on a variety of factors such as particle geometry, optical characteristics of suspended material (i.e., reflectance, color, absorption, transmittance), and equipment used to measure turbidity (Hannouche et al. 2011). The correlation between turbidity and TSS is often very strong, linked by a power function (Perkins et al. 2014; Shen et al. 2018). The power function showed the strongest fit (*R*^2^ of 0.98) compared to linear and other non-linear models (e.g., logarithmic, exponential, polynomial). Therefore, this consistent relationship could serve as a proxy for estimating TSS in construction site runoff.

### Particle size distribution

**Fig. 5:**
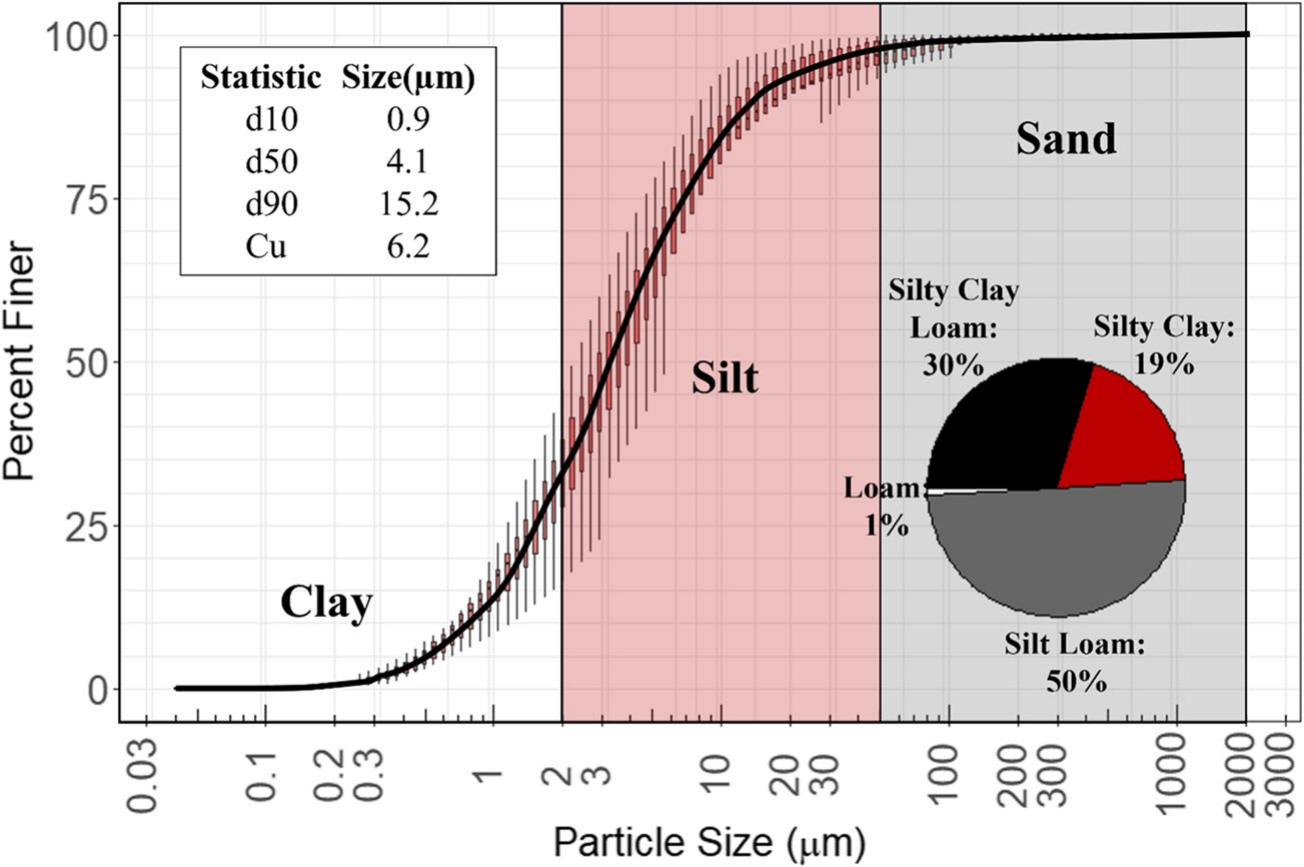
Aggregated particle size distribution for the 104 PSD samples. Each boxplot represents the variability in particle size for that particle size class. The pie chart in the bottom right corner shows the percentage of textural classes across all samples

An aggregated PSD formed by connecting the median particle size from all collected samples (*n* = 104) is presented in Fig. 5. Median d_10_, d_50_, and d_90_ particle diameters across all samples were 0.9 μm, 4.1 μm, and 15.2 μm, respectively. The median coefficient of uniformity across all sites was greater than four, suggesting that most of the suspended sediments were well graded (Table 3). Unlike TSS and turbidity, relatively small variations existed in the d_50_ particle diameter between each construction site, with d_50_ values ranging from 2.9–5.1 μm, respectively (Table 3). However, the median d_90_ particle diameter was more variable, ranging from 8.8–23.6 μm. Nonetheless, median d_90_ particle diameters were found to be in the silt fraction at all three construction sites (Table 3).

**Table 3:** Median summary statistics for PSD by monitoring site

Site	Median ± MAD (range)			
	d10	d50	d90	*C*_*u*_
One	0.8 ± 0.4 μm (0.4–1.3)	3.8 ± 1.9 μm (2.5–10.7)	15.2 ± 10.9 μm (5.5–107.1)	6.6 ± 3.6 (4.6–12.4)
Two	1.1 ± 0.1 μm (0.5–7.8)	5.1 ± 1.8 μm (2.1–30.8)	23.6 ± 15.2 μm (6.1–483.8)	6.5 ± 1.9 (3.9–25.6)
Three	0.8 ± 0.1 μm (0.6–1.1)	2.9 ± 0.7 μm (1.8–7.8)	8.8 ± 2.6 μm (5.3–74.9)	4.7 ± 1 (3.4–10.8)

Half of the samples had a soil textural class of silt loam; the remaining samples were characterized by silty clay loam (30%), silty clay (19%), and loam (1%) textural classifications (Fig. 5). The high proportions of silt and clay are typical of subsoils in Ohio (McCormack and Wilding 1969), suggesting that excavation of topsoil exposed soil B horizons to erosion. Conversely, Winston et al. (2023) found a median d_50_ of 52.5 μm across 176 sampled storms from Ohio roads, implying that post-construction runoff contained coarser sediments. Due to the prevalence of finer particles (i.e., silt and clay) in this study, substantial sediment (and associated sediment-bound pollutants) removal may be challenging for ESCMs that rely on sedimentation as the primary pollutant removal mechanism. One potential solution to this issue is the use of flocculants (e.g., polyacrylamide and chitosan), polymers commonly used in wastewater treatment and at construction sites to agglomerate suspended particles into larger particles for quicker settling. In construction site runoff applications, flocculants have proven reliable at reducing turbidity and shifting PSD into coarser fractions (McLaughlin et al. 2009; Kang et al. 2013, 2014).

### Relationship between TSS, turbidity, PSD, and rainfall characteristics

**Fig. 6:**
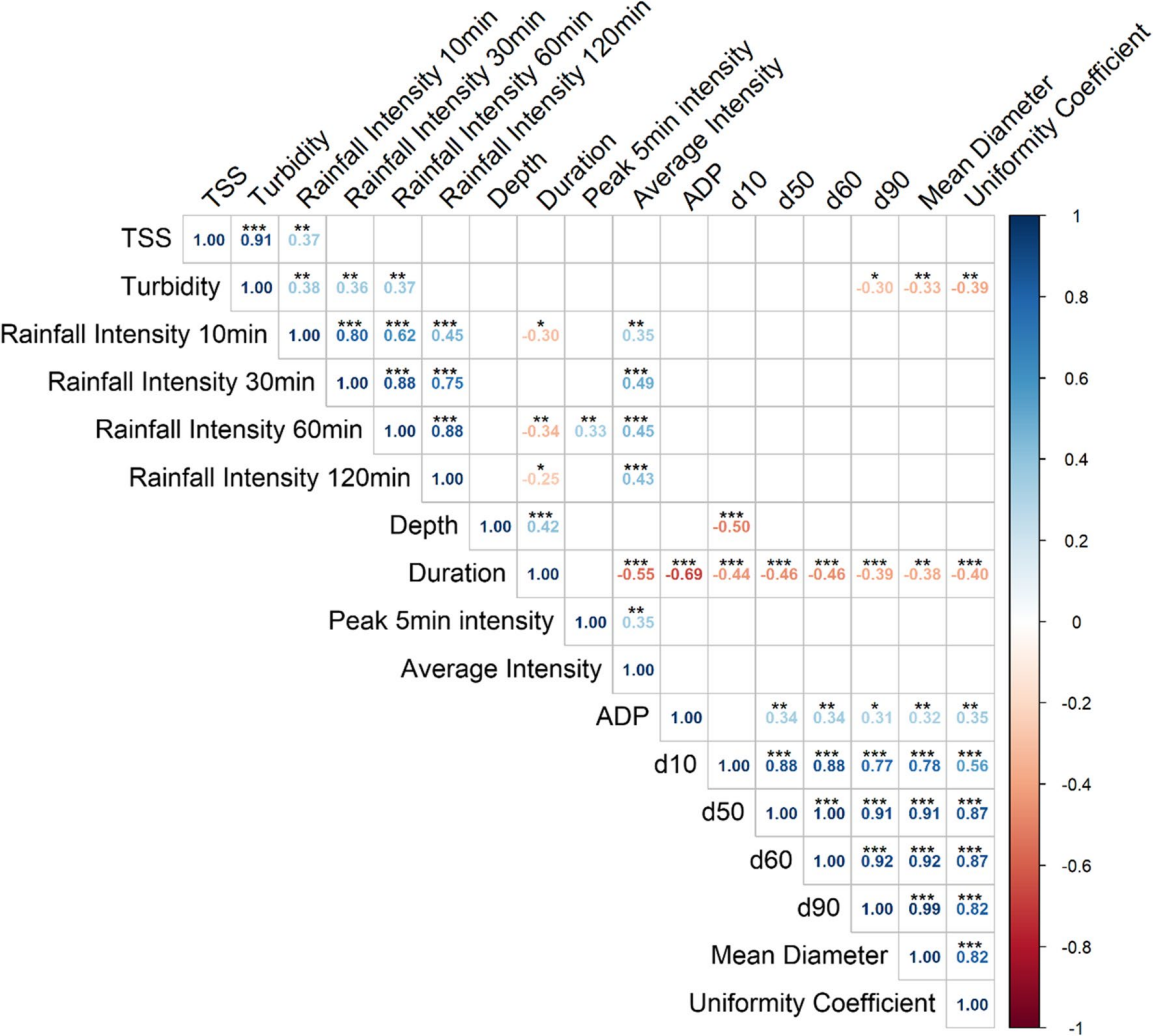
Correlogram between TSS, turbidity, particle size statistics (d_10_, d_50_, d_60_, d_90_, mean diameter, *C*_*u*_), and rainfall characteristics (depth, duration, preceding rainfall intensity 10, 30, 60, and 120 min prior to obtaining a grab sample, peak 5 min and average rainfall intensity, and antecedent dry period). The number of asterisks (i.e., ***, **, and *) refers to *p-*values less than 0.001, 0.01, and 0.05, respectively

Significant positive correlations were observed between the rainfall intensity 10, 30, and 60 min prior to obtaining a grab sample and turbidity (Fig. 6). There is consensus that rainfall intensity is proportional to the erosive potential of rain events, with higher rainfall intensities and resultant flow rates creating greater shear stress on soils (Watson and Laflen 1986). The 10-min rainfall intensity prior to sample collection was the most strongly correlated rainfall characteristic with TSS and turbidity across all sites (Fig. 6). However, TSS concentrations were not significantly correlated to the 30- and 60-min rainfall intensities prior to sample collection. This could be attributed to the relatively small times of concentration (i.e., less than 5 min) observed at each monitoring location, suggesting that the rainfall intensity closest to the time of sample collection had the largest impact on sediment mobilization. However, for some sites (i.e., G, H, and J), the 60-min intensities prior to sample collection had larger correlations compared to the 10-min intensity, alluding to those sites that may have had longer times of concentration (see supplementary materials Figs. 2A-4A).

Significant negative correlations were observed between both rainfall depth and duration with particle size parameters, indicating that larger rainfall depths and longer rainfall durations resulted in finer particle sizes in construction site runoff. Soil aggregates tend to be weaker in wetter soils (Le Bissonnais 1996), implying that larger or longer events which result in saturated conditions promote the dispersion of soil aggregates. Conversely, ADP was positively correlated with particle size parameters and average rainfall intensity, implying that drier periods and higher rainfall intensities caused coarser particles to mobilize during rainfall. It is possible that drier soils may be more susceptible to erosion from reduced cohesion and water-holding capacity (Moragoda et al. 2022). Moreover, drier soils are prone to slaking when impacted by rainfall, reducing porosity due to microaggregates filling pore spaces (Lal and Shukla 2004).

### Multivariable linear regression analysis

Multivariable linear regression analysis using backward selection resulted in the following model to predict TSS concentrations:

3 $$\mathrm{TSS }= 0.72 \times {\mathrm{ intensity}}_{10{\text{min}}} - 0.44 \times {{\text{intensity}}}_{30{\text{min}}} + 0.69 \times {\mathrm{ intensity}}_{60{\text{min}}} - 0.97 \times {\mathrm{ intensity}}_{120{\text{min}}} - 0.67 \times \mathrm{ depth }+ 0.51 \times \mathrm{ duration }+ 0.86 \times {{\text{intensity}}}_{{\text{avg}}}$$

where TSS is in milligrams per liter; intensity_10min_, intensity_30min_, intensity_60min_, and intensity_120min_ are the rainfall intensities (mm/h) 10, 30, 60, and 120 min prior to sample collection, respectively; depth is the event depth (mm); duration is the event duration (h); and intensity_avg_ is the average rainfall intensity (mm/h) (*p* < 0.01, *R*^2^ = 0.50). Model results suggest that higher TSS concentrations are associated with greater rainfall intensities, depths, and durations. Further, similar methods were used to develop the following relationship between predictor variables and turbidity:

4 $$\mathrm{Turbidity }= 1.10 \times {{\text{intensity}}}_{10{\text{min}}} - 0.88 \times {{\text{intensity}}}_{30{\text{min}}} + 0.26 \times {{\text{intensity}}}_{{\text{Avg}}}$$

where turbidity is in NTU, intensity_10min_ and intensity_30min_ are the rainfall intensities 10 and 30 min prior to sample collection (mm/h), and intensity_avg_ is the average rainfall intensity (mm/h) (*p* < 0.01, *R*^2^ = 0.37). Similar to the model for TSS, higher rainfall intensities resulted in greater turbidity values.

### Pilot study of sediment pollutograph

**Fig. 7:**
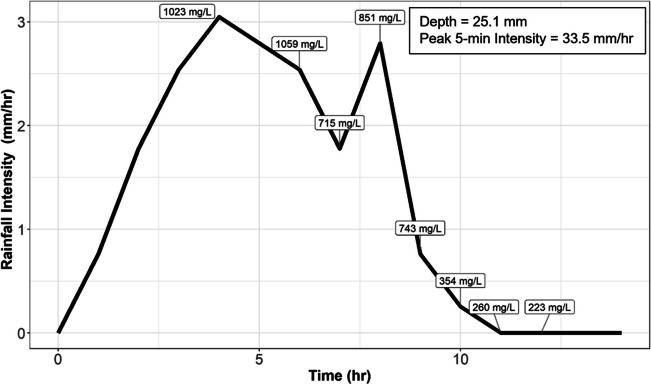
Time series of hourly rainfall intensity and TSS for a storm event on 11–25-2020 at monitoring location H

Hourly rainfall intensity was highly correlated (ρ = 0.9) with TSS during high-frequency sampling at monitoring location H (Fig. 7), with higher rainfall intensities corresponding to larger TSS concentrations. Similar relationships were observed for monitoring locations G and J (see supplementary material Figs. 5A and 6A). TSS varied considerably over the course of the event (range 223–1059 mg/L). Similar variability was observed for monitoring locations G (range 1179–3395 mg/L) and J (range 320–1033 mg/L). This suggests that ESCMs may need to be designed for relatively high rainfall intensities as these appear to be driving a substantial portion of the sediment transport.

## Conclusions

Runoff from three active construction sites in central Ohio was sampled and analyzed for TSS, turbidity, and PSD. Water quality parameters (e.g., TSS, turbidity, and PSD) and rainfall characteristics were analyzed to identify factors influencing sediment export from construction sites. The following conclusions can be drawn from this research:

1. Substantial variability in TSS and turbidity can be expected in construction site runoff. The range of TSS and turbidity across all samples were 25–28,600 mg/L (median of 626 mg/L ± MAD 426 mg/L) and 22–33,000 NTU (median of 759 NTU ± MAD 541 NTU), respectively. Conversely, little variation existed in the d_50_ across samples, with soil textural classes from collected samples mimicking typical subsoils in Central Ohio (i.e., silt loam). Considering the wide range of TSS and turbidity observed, ESCMs must be resilient in their design to withstand variable sediment loading during rainfall. Additionally, ESCM design should consider subsoil PSDs to ensure proper hydraulic retention time or other mechanisms for sediment retention.
2. The rainfall intensity, 10, 30, and 60 min, prior to sample collection was significantly positively correlated with turbidity, implying that intensity impacted sediment generation on the studied construction sites. The rainfall intensity 10 min prior to sample collection was most correlated to TSS and turbidity across all samples, most likely due to the relatively short times of concentration for each sampling location. A higher frequency sampling effort conducted over the course of a single hyetograph confirmed this significant positive relationship. Therefore, reducing raindrop impact associated with higher rainfall intensities is crucial to mitigating sediment export from construction sites.
3. Despite minimal variability in particle size among samples, longer rainfall durations led to overall smaller PSD. This was attributed to saturated soil conditions and the dispersion of larger soil aggregates. Additionally, ADP was positively correlated with average rainfall intensity and particle size. This may be related to the mobilization of larger particles following dry conditions paired with more intense rainfall. Thus, soil moisture conditions should be considered when designing ESCMs.

### Supplementary Information

Below is the link to the electronic supplementary material.

Supplementary file1 (DOCX 3103 KB)

## Data Availability

Raw data related to this study are available from the corresponding author, upon reasonable request.

## References

[CR1] Amundson R, Guo Y, Gong P (2003). Soil diversity and land use in the United States. Ecosystems.

[CR2] Arjmand Sajjadi S, Mahmoodabadi M (2015). Aggregate breakdown and surface seal development influenced by rain intensity, slope gradient and soil particle size. Solid Earth.

[CR3] ASTM D5907–18 (2018) Standard test methods for filterable matter (total dissolved solids) and non-filterable matter (total suspended solids) in water. American Society for Testing and Materials (ASTM), West Conshohocken, PA

[CR4] Barron MG (1995). Bioaccumulation and bioconcentration in aquatic organisms. Handbook of Ecotoxicology.

[CR5] Binns AD, Fata A, Ferreira da Silva AM, Bonakdari H, Gharabaghi B (2019). Modeling performance of sediment control wet ponds at two construction sites in Ontario. Canada J Hydraul Eng.

[CR6] Blecken GT, Hunt WF, Al-Rubaei AM, Viklander M, Lord WG (2017). Stormwater control measure (SCM) maintenance considerations to ensure designed functionality. Urban Water J.

[CR7] Brown LC, Foster GR (1987). Storm erosivity using idealized intensity distributions. Trans ASAE.

[CR8] Bugg R, Donald W, Zech W, Perez M (2017). Performance evaluations of three silt fence practices using a full-scale testing apparatus. Water.

[CR9] Charters FJ, Cochrane TA, O'Sullivan AD (2015). Particle size distribution variance in untreated urban runoff and its implication on treatment selection. Water Res.

[CR10] Clark SE, Allison AA, Sitler RA (2009). Geographic variability of rainfall erosivity estimation and impact on construction site erosion control design. J Irrig Drain Eng.

[CR11] Crowder BM (1987). Economic costs of reservoir sedimentation: a regional approach to estimating cropland erosion damage. J Soil Water Conserv.

[CR12] Daniel TC, Mc Guire PE, Stoffel D, Miller B (1979). Sediment and nutrient yield from residential construction sites. J Environ Qual.

[CR13] Ebrahimzadeh S, Motagh M, Mahboub V, Mirdar Harijani F (2018). An improved RUSLE/SDR model for the evaluation of soil erosion. Environ Earth Sci.

[CR14] Fang X, Zech WC, Logan CP (2015). Stormwater field evaluation and its challenges of a sediment basin with skimmer and baffles at a highway construction site. Water.

[CR15] Fiksen Ø, Aksnes DL, Flyum MH, Giske J (2002) The influence of turbidity on growth and survival of fish larvae: a numerical analysis. In: Sustainable increase of marine harvesting: fundamental mechanisms and new concepts: proceedings of the 1st Maricult Conference. Trondheim, Norway, pp 49–59

[CR16] Furumai H, Balmer H, Boller M (2002). Dynamic behavior of suspended pollutants and particle size distribution in highway runoff. Water Sci Technol.

[CR17] Greb SR, Bannerman RT (1997). Influence of particle size on wet pond effectiveness. Water Environ Res.

[CR18] Hannouche A (2011). Relationship between turbidity and total suspended solids concentration within a combined sewer system. Water Sci Technol.

[CR19] Hilliges R, Endres M, Tiffert A, Brenner E, Marks T (2017). Characterization of road runoff with regard to seasonal variations, particle size distribution and the correlation of fine particles and pollutants. Water Sci Technol.

[CR20] Kang J, McCaleb MM, McLaughlin RA (2013). Check dam and polyacrylamide performance under simulated stormwater runoff. J Environ Manage.

[CR21] Kang J, King SE, McLaughlin RA (2014). Impacts of flocculation on sediment basin performance and design. Trans ASABE.

[CR22] Kayhanian M, Murphy K, Regenmorter L, Haller R (2001). Characteristics of storm-water runoff from highway construction sites in California. Transp Res Rec J Transp Res Board.

[CR23] Keener HM, Faucette B, Klingman MH (2007). Flow-through rates and evaluation of solids separation of compost filter socks versus silt fence in sediment control applications. J Environ Qual.

[CR24] Kiani-Harchegani M, Sadeghi SH, Singh VP, Asadi H, Abedi M (2019). Effect of rainfall intensity and slope on sediment particle size distribution during erosion using partial eta squared. CATENA.

[CR25] Kinnell PIA (1973). The problem of assessing the erosive power of rainfall from meteorological observations. Soil Sci Soc Am J.

[CR26] Lal R, Shukla MK (2004). Principles of soil physics.

[CR27] Lalor M, Angus R, Marion K, Owens J, Clark S (2004) Effectiveness of current BMPs in controlling sediment discharges from small construction sites. In: Proceedings of the WEFTEC conference. Water Environment Federation, pp 529–540

[CR28] Langston WJ (2010). Contaminants in fine sediments and their consequences for biota of the Severn Estuary. Mar Pollut Bull.

[CR29] Le Bissonnais YL (1996). Aggregate stability and assessment of soil crustability and erodibility: I. Theory and methodology. Eur J Soil Sci.

[CR30] Li Y, Lau SL, Kayhanian M, Stenstrom MK (2005). Particle size distribution in highway runoff. J Environ Eng.

[CR31] Line DE, White NM (2007). Effects of development on runoff and pollutant export. Water Environ Res.

[CR32] Line DE, White NM, Osmond DL, Jennings GD, Mojonnier CB (2002). Pollutant export from various land uses in the Upper Neuse River Basin. Water Environ Res.

[CR33] Line DE, Shaffer MB, Blackwell JD (2011). Sediment export from a highway construction site in central North Carolina. Trans ASABE.

[CR34] Lloyd DS, Koenings JP, Laperriere JD (1987). Effects of turbidity in fresh waters of Alaska. North Am J Fish Manag.

[CR35] Lunt J, Smee DL (2014). Turbidity influences trophic interactions in estuaries. Limnol Oceanogr.

[CR36] Martínez-Mena M, Castillo V, Albaladejo J (2002). Relations between interrill erosion processes and sediment particle size distribution in a semiarid Mediterranean area of SE of Spain. Geomorphology.

[CR37] McCormack DE, Wilding LP (1969). Variation of soil properties within mapping units of soils with contrasting substrata in Northwestern Ohio. Soil Sci Soc Am J.

[CR38] McLaughlin RA, Hayes SA, Clinton DL, McCaleb MS, Jennings GD (2009). Water quality improvements using modified sediment control systems on construction sites. Trans ASABE.

[CR39] Moragoda N, Kumar M, Cohen S (2022). Representing the role of soil moisture on erosion resistance in sediment models: challenges and opportunities. Earth-Sci Rev.

[CR40] Nighman D, Harbor J (1997). Trap efficiency of a stormwater basin with and without baffles. Proc Int Erosion Control Assn.

[CR41] Perez MA, Zech WC, Donald WN, Fang X (2015). Methodology for evaluating inlet protection practices using large-scale testing techniques. J Hydrol Eng.

[CR42] Perkins R, Hansen B, Wilson B, Gulliever J (2014) Development and evaluation of effective turbidity monitoring methods for construction projects. In: Minnesota Department of Transportation Final Project Report 2014–24. Minnesota Dept. of Transportation Research Services & Library, St. Paul, MN

[CR43] Pitt R, Clark SE, Lake DW (2007) Construction site erosion and sediment controls: planning, design and performance. DEStech Publications, Inc., Lancaster, PA

[CR44] R Core Team (2019) R: a language and environment for statistical computing. R Foundation for Statistical Computing, Vienna, Austria. Available at: http://www.Rproject.org

[CR45] Rahmani V, Kastens JH, DeNoyelles F, Jakubauskas ME, Martinko EA, Huggins DH, Blackwood AJ (2018). Examining storage capacity loss and sedimentation rate of large reservoirs in the central US Great Plains. Water.

[CR46] Renard KG, Ferreira VA (1993). RUSLE model description and database sensitivity. J Environ Qual.

[CR47] Römkens MJ, Helming K, Prasad SN (2002). Soil erosion under different rainfall intensities, surface roughness, and soil water regimes. CATENA.

[CR48] Sansalone JJ, Koran JM, Smithson JA, Buchberger SG (1998). Physical characteristics of urban roadway solids transported during rain events. J Environ Eng.

[CR49] Santikari VP, Murdoch LC (2019). Effects of construction-related land use change on streamflow and sediment yield. J Environ Manage.

[CR50] Schussler JC, Perez MA, Whitman JB, Cetin B (2022). Field-monitoring sediment basin performance during highway construction. Water.

[CR51] Schussler J, Perez M, Cetin B, Whitman J (2020) Field monitoring of erosion and sediment control practices and development of additional Iowa DOT Design Manual Guidance. Final Report 18-654. Iowa Department of Transportation, Ames, IA

[CR52] Sear DA (1996). Sediment transport processes in pool–riffle sequences. Earth Surf Process Landf.

[CR53] Selbig WR, Fienen MN, Horwatich JA, Bannerman RT (2016). The effect of particle size distribution on the design of urban stormwater control measures. Water.

[CR54] Shen C, Liao Q, Titi HH, Li J (2018). Turbidity of stormwater runoff from highway construction sites. J Environ Eng.

[CR55] Simpson IM, Winston RJ, Brooker MR (2022). Effects of land use, climate, and imperviousness on urban stormwater quality: a meta-analysis. Sci Total Environ.

[CR56] Smith PM (2018) Monitoring and assessment of sediment basins at highway construction sites. Master's Thesis, Environmental Engineering, University of Tennessee

[CR57] Tirpak RA, Winston RJ, Simpson IM, Dorsey JD, Grimm AG, Pieschek RL, Petrovskis EA, Carpenter DD (2021). Hydrologic impacts of retrofitted low impact development in a commercial parking lot. J Hydrol.

[CR58] U.S. Environmental Protection Agency (USEPA), Office of Water (2000) Stormwater phase II final rule: construction site runoff control minimum control measure. Publication EPA 833/F-00/008. Fact Sheet 2.6. Washington, DC

[CR59] U.S. Geological Survey (USGS) (2016) National Water Information System data. Available at: http://waterdata.usgs.gov/nwis/

[CR60] Walling DE, Gregory KJ (1970). The measurement of the effects of building construction on drainage basin dynamics. J Hydrol.

[CR61] Watson DA, Laflen JM (1986). Soil strength, slope, and rainfall intensity effects on interrill erosion. Trans ASAE.

[CR62] Winston RJ, Hunt WF (2017). Characterizing runoff from roads: particle size distributions, nutrients, and gross solids. J Environ Eng.

[CR63] Winston RJ, Al-Rubaei AM, Blecken GT, Viklander M, Hunt WF (2016). Maintenance measures for preservation and recovery of permeable pavement surface infiltration rate–the effects of street sweeping, vacuum cleaning, high pressure washing, and milling. J Environ Manage.

[CR64] Winston RJ, Witter JD, Tirpak RA (2023). Evaluating sediment load and particle size distribution in road runoff: Implications for sediment removal by stormwater control measures.

[CR65] Wischmeier WH, Smith DD (1958). Rainfall energy and its relationship to soil loss. Trans Am Geophys Union.

[CR66] Wolman MG, Schick AP (1967). Effects of construction on fluvial sediment, urban and suburban areas of Maryland. Water Resour Res.

[CR67] Wood PJ, Armitage PD (1997). Biological effects of fine sediment in the lotic environment. Environ Manage.

[CR68] Zech WC, Logan CP, Fang X (2014). State of the practice: evaluation of sediment basin design, construction, maintenance, and inspection procedures. Pract Period Struct Des Constr.

