# Supplementary material for: Monitoring the impacts of rainfall characteristics on sediment loss from road construction sites
Source: Environ Sci Pollut Res Int. 2024 Apr 23;31(22):32428–40. doi: 10.1007/s11356-024-33361-3 (PMC11133084; doi:10.1007/s11356-024-33361-3)
Supplement: Supplementary file 1 — Supplementary file1 (DOCX 3103 KB) [file 11356_2024_33361_MOESM1_ESM.docx]

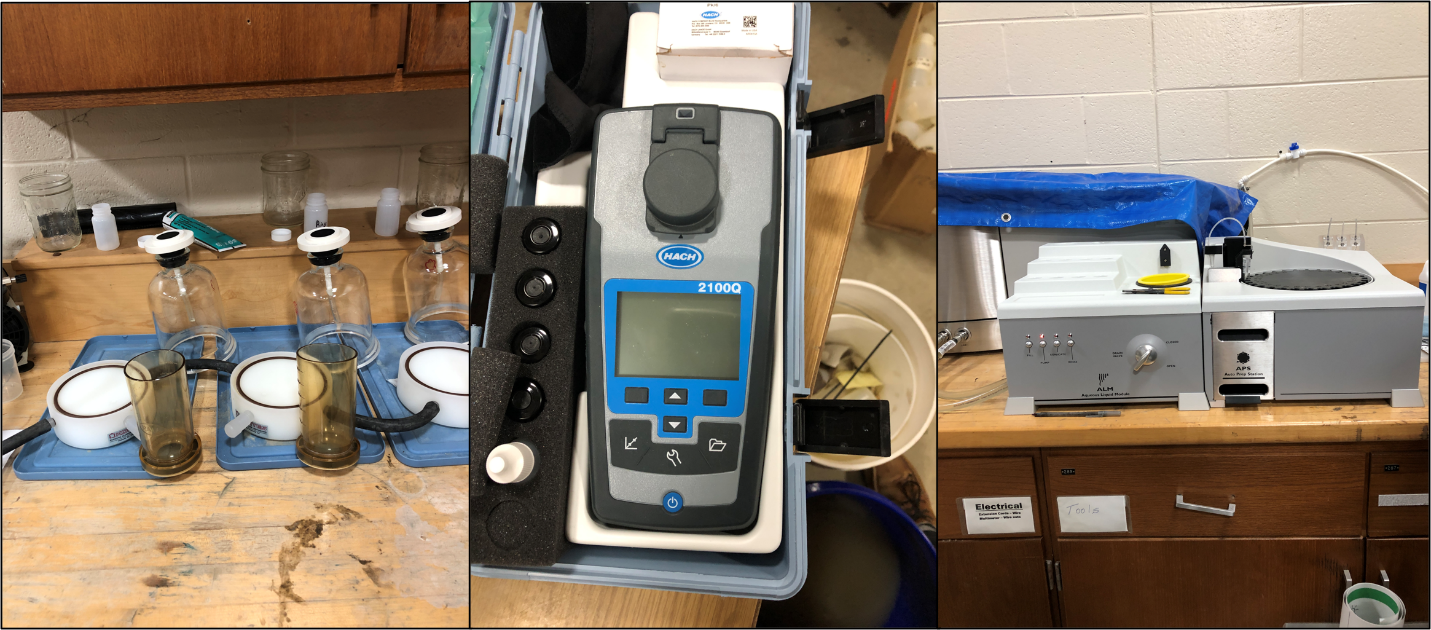


Figure 1A: Photos of vacuum filtration for TSS determination, turbidimeter, and laser diffraction particle size analyzer, respectively.


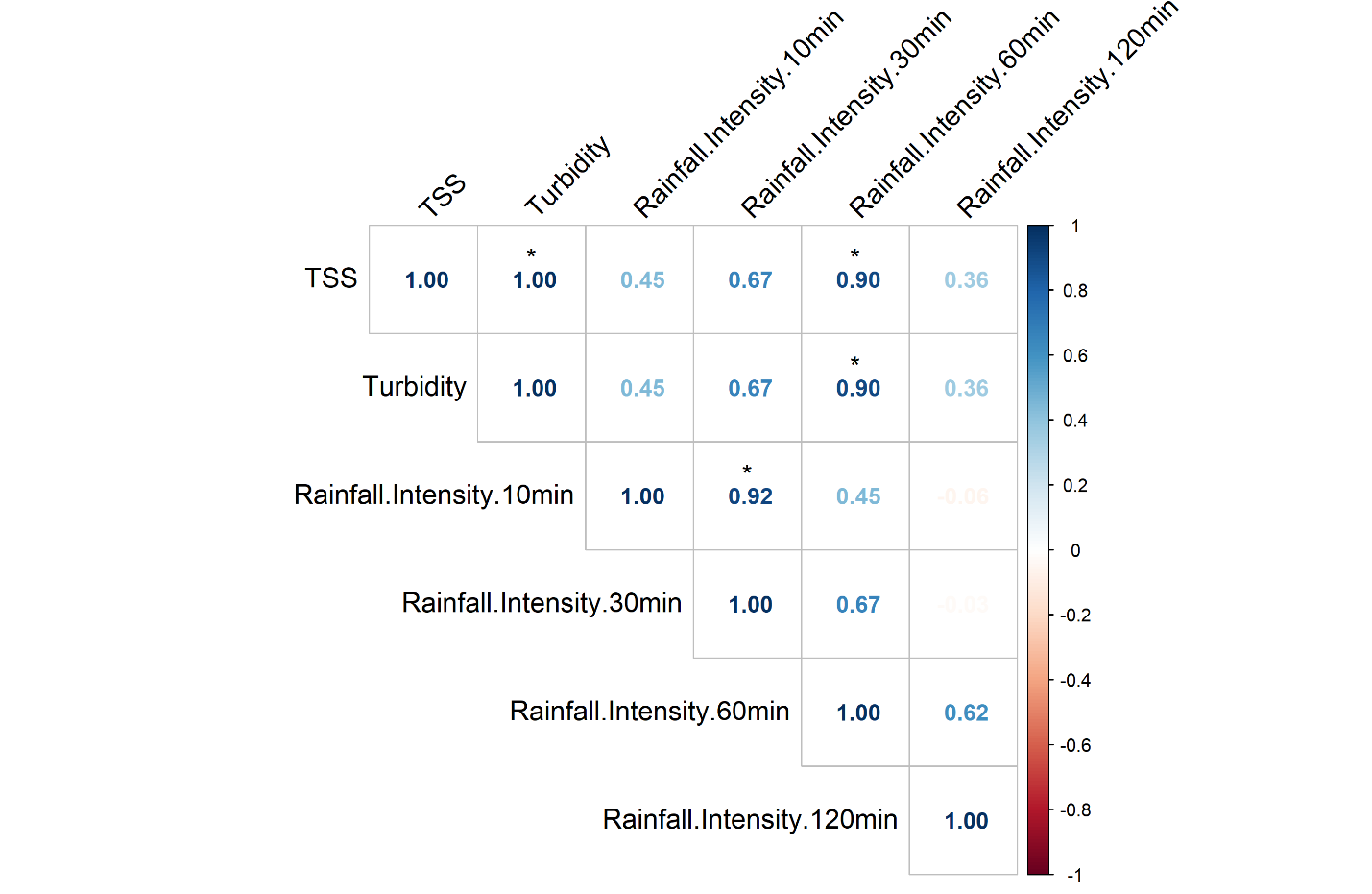


Figure 2A: Correlogram between TSS, turbidity and rainfall intensities for monitoring location G. Asterisk represents correlations that are statistically significant.


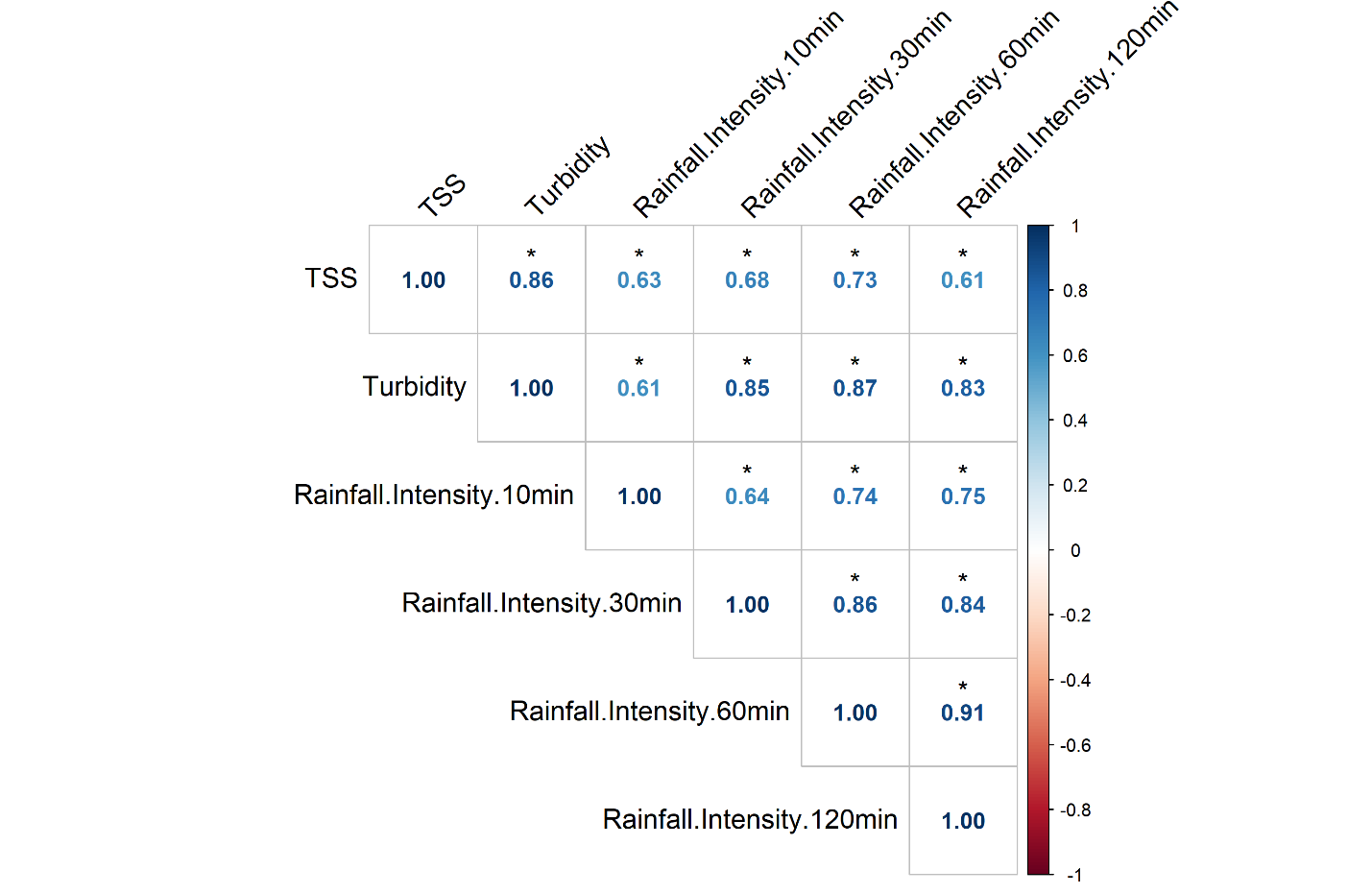


Figure 3A: Correlogram between TSS, turbidity and rainfall intensities for monitoring location H. Asterisk represents correlations that are statistically significant.


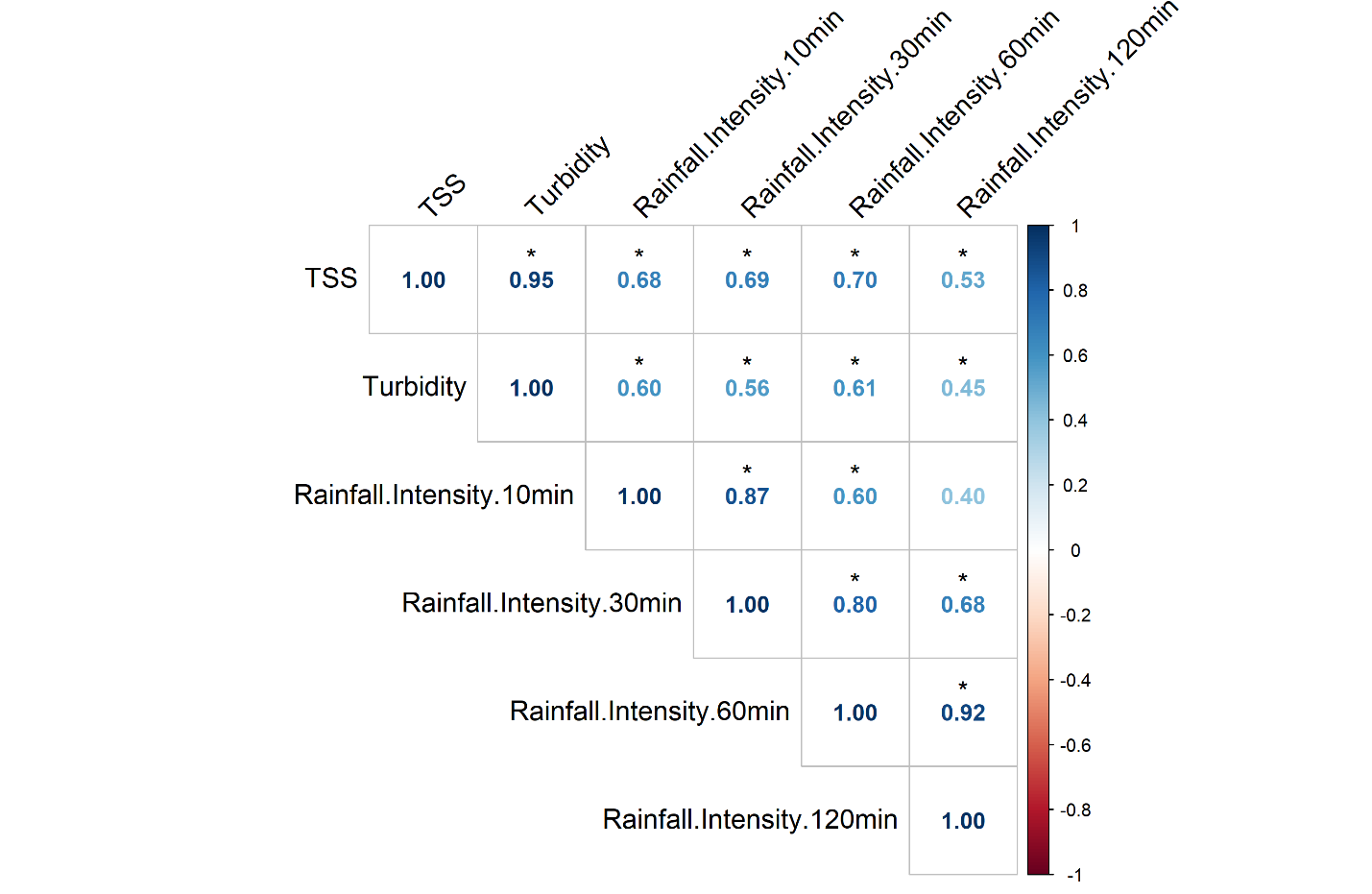


Figure 4A: Correlogram between TSS, turbidity and rainfall intensities for monitoring location J. Asterisk represents correlations that are statistically significant.


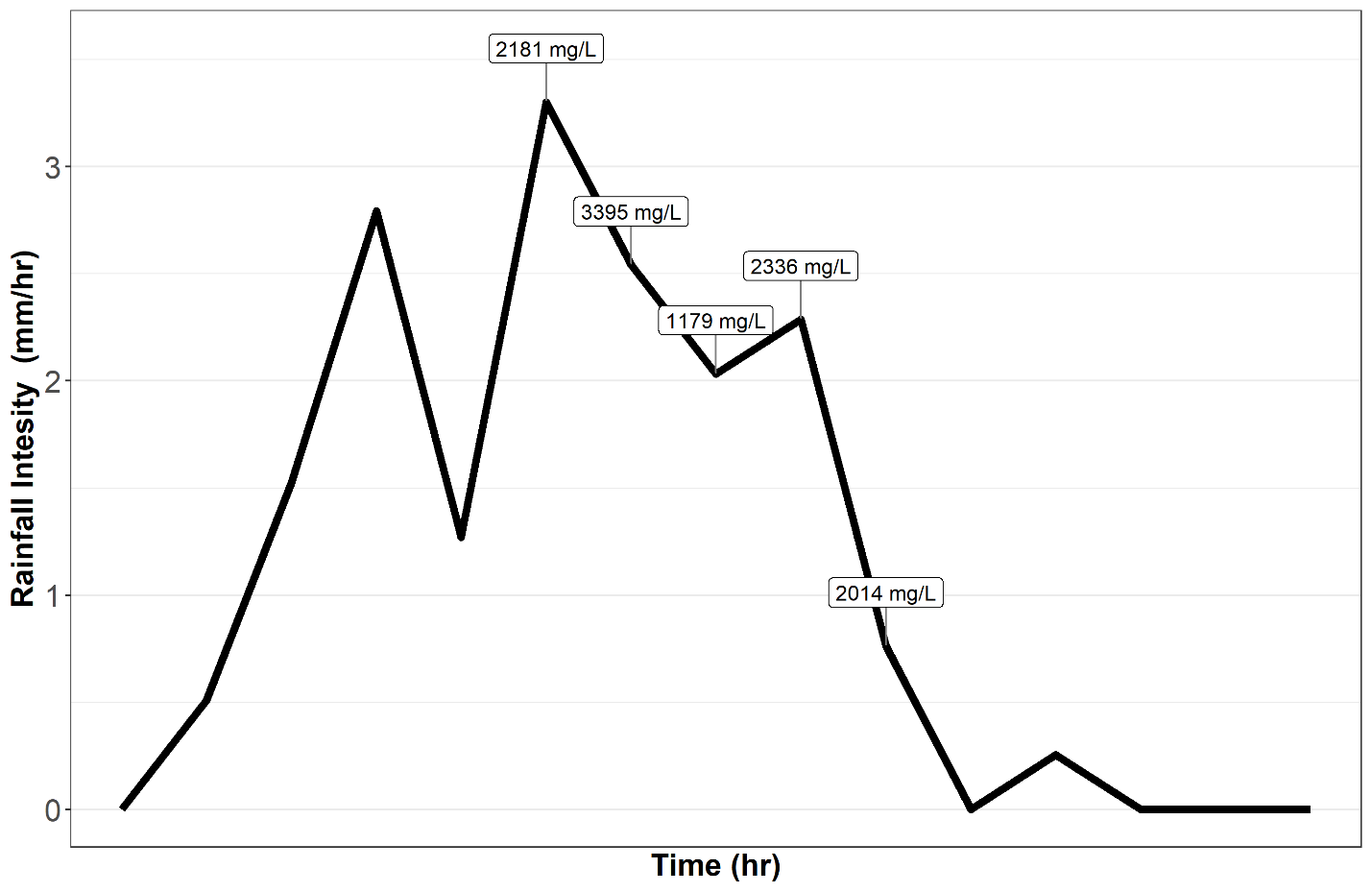


Figure 5A: Time series of hourly rainfall intensity and TSS for a storm event on 11-25-2020 at monitoring location G.


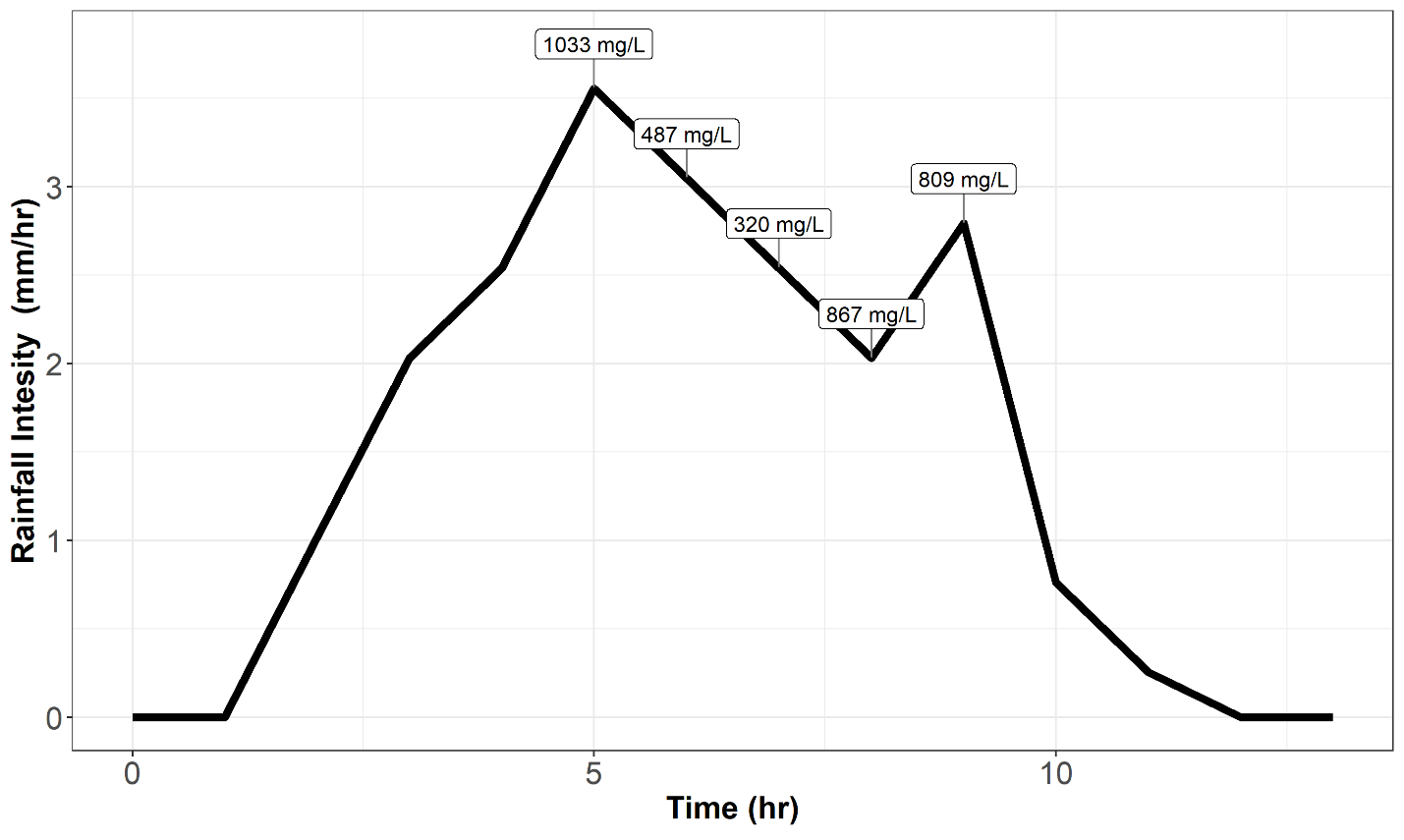


Figure 6A: Time series of hourly rainfall intensity and TSS for a storm event on 11-25-2020 at monitoring location J.
